# Luminescent Lifetime Regulation of Lanthanide-Doped Nanoparticles for Biosensing

**DOI:** 10.3390/bios12020131

**Published:** 2022-02-19

**Authors:** Mingkai Wang, Chuanyu Hu, Qianqian Su

**Affiliations:** 1 Department of Stomatology, Tongji Hospital, Tongji Medical College, Huazhong University of Science and Technology, Wuhan 430074, China; wmk19961108@163.com; 2 Institute of Nanochemistry and Nanobiology, Shanghai University, Shanghai 200444, China

**Keywords:** luminescence lifetime, lanthanide-doped nanoparticles, lifetime regulation, bioimaging, biodetection, biosensing

## Abstract

Lanthanide-doped nanoparticles possess numerous advantages including tunable luminescence emission, narrow peak width and excellent optical and thermal stability, especially concerning the long lifetime from microseconds to milliseconds. Differing from other shorter-lifetime fluorescent nanomaterials, the long lifetime of lanthanide-doped nanomaterials is independent with background fluorescence interference and biological tissue depth. This review presents the recent advances in approaches to regulating the lifetime and applications of bioimaging and biodetection. We begin with the introduction of the strategies for regulating the lifetime by modulating the core–shell structure, adjusting the concentration of sensitizer and emitter, changing energy transfer channel, establishing a fluorescence resonance energy transfer pathway and changing temperature. We then summarize the applications of these nanoparticles in biosensing, including ion and molecule detecting, DNA and protease detection, cell labeling, organ imaging and thermal and pH sensing. Finally, the prospects and challenges of the lanthanide lifetime regulation for fundamental research and practical applications are also discussed.

## 1. Introduction

A central goal in biology and medicine is to develop excellent imaging and detection technology [1]. Researchers have devoted massive efforts to developing fluorescence imaging technology through fluorescent nanomaterials, including quantum dots (QDs) [2], upconversion nanomaterials (UCNPs) [3], carbon-based nanomaterials [4], stoichiometric metal oxides [5], perovskite [6], metal nanomaterials [7] and other materials [8]. In addition, biological images were obtained in a non-invasive manner under laser excitation [9], which has great labeling ability, high signal strength, fast imaging speed and a wide range of applications [10,11,12,13]. However, there are numerous limitations based on conventional fluorescence imaging technology, including low sensitivity, shallow detective depth and substantial interference caused by background autofluorescence [14,15]. Among the large number of nanomaterials, lanthanide-doped nanoparticles feature a longer lifetime than other fluorescence materials, whose emission could last for milliseconds [16]. Based on these nanoparticles, lifetime imaging and detection platform were proposed to resolve the difficulty of conventional fluorescence imaging [17,18].

Unsusceptible to the concentration of nanoparticles [19], laser intensity [20] and biological tissue thickness [21], the quality of luminescence lifetime imaging is better than traditional luminescence imaging in the cell or tissue microenvironment [22]. Moreover, the sensitivity of time-gated imaging with an optical chopper offers more than one order of magnitude than that of the traditional method using optical band-pass filters [23]. The luminescence lifetime of lanthanides depends on their intrinsic properties and the localized microenvironment, involving pH value, doped ion concentration, protein interaction and other factors affecting the decay process [24]. Therefore, lifetime encoding was achieved by nanostructural engineering, while lifetime sensing was produced by changing the relying circumstances. Correspondingly, the time-resolved technology was developed rapidly due to its unique properties [25].

**Figure 1:**
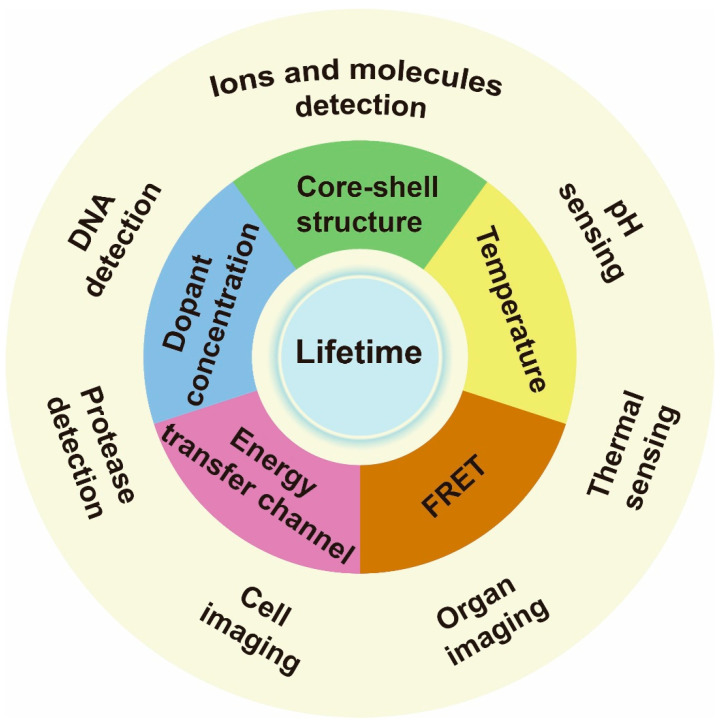
Schematic illustrations of lanthanide-doped nanoparticle lifetime regulation for biosensing. The approaches to regulating lifetime include varying core–shell structure, dopant concentration, energy transfer channel, temperature and setting a fluorescence resonance energy transfer (FRET) system. The relevant applications include ions, molecules, DNA, protease, thermal and pH sensing, as well as cell labeling and organ imaging.

In this review, we focus on the recent advances in the regulating lifetime of lanthanide for biosensing and imaging (Figure 1). In Section 1, we introduce the strategies of lifetime modulation for lanthanide-doped nanoparticles, involving core–shell structure design, concentration adjusting, energy transfer channel controlling and temperature controlling. In Section 2, we discuss the use of the probes for ions and molecules, DNA and protease sensing, cell labeling, organ imaging and thermal and pH sensing. Finally, we discuss the prospects and challenges for possible development directions and application scenarios.

## 2. Lifetime Regulation

Luminescence lifetime refers to the average time the molecule spends in the excited state returning to the ground state [26]. The lifetime of a phosphor, τ, refers to the time at which the intensity decays to 1/e of its maximum [27]. The decay of one emitting state in an upconversion energy transfer process is determined by its intrinsic decay and the intermediate states [28]. Susceptive to their intrinsic properties and local environment, the lifetime of lanthanide-doped nanoparticles could be tuned by adjusting core–shell structure, the content of sensitizer and emitter, internal energy transfer channel, FRET system and temperature. Nanocrystals with a controllable lifetime have been widely used in biosensing to eliminate background autofluorescence and light scattering interference. However, the reported methods allow only a limited range of lifetime adjustments.

### 2.1. Variation of Core–Shell Structures

Core–shell engineering is generally utilized to reduce surface quenching effects [29]. Shell passivation could promote the upconversion process by promoting the occurrence of energy hopping in higher-lying excited states [30]. Therefore, lifetime is affected by the shell layers, including the shell thickness and shell composition.

#### 2.1.1. Core Size

**Figure 2:**
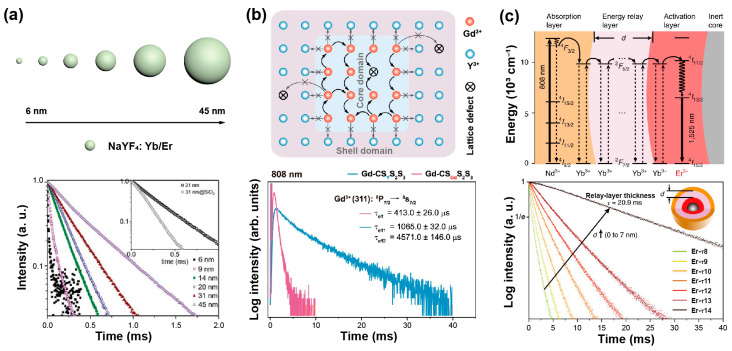
(**a**) The schematic description for the size of NaYF_4_: Yb,Er nanoparticles and lifetime decay curves of green emission in these nanoparticles. Reproduced with permission from [31]. Copyright 2013, Royal Society of Chemistry. (**b**) The diagram of energy transfer mechanism in NaGdF_4_: 49%Yb,1%Tm coated with heterogeneous shell nanoparticle, and corresponding lifetimes of Gd^3+^ in homogenerous and heterogeneous nanoparticles at 310 nm under 808 nm excitation. Reproduced with permission from [38]. Copyright 2021, Nature Publishing Group. (**c**) The diagrammatic drawing of energy transfer process in the core–multishell nanoparticles and luminescence decay curves at 1525 nm with the energy relay shell thickness ranging from 0 nm to 7 nm. Reproduced with permission from [43]. Copyright 2018, Nature Publishing Group.

The defect density of core nanoparticles doped with sensitizers and emitters decreases as the nanoparticle’s size increases. Jin et al. found an increase in the lifetime of Yb, Er-doped UCNPs with the increase of their size by comparing the lifetime of these nanoparticles ranging from 6 nm to 45 nm (Figure 2a). With the assistance of a mathematical model, it was verified that the decay time was related to surface-to-volume ratio and shell thickness. In addition, the lifetime of β-phase particles has significant relationships with surface defect, solvent and vibration energy of surface ligands, while defect density only matters for α-phase nanocrystals [31]. Liu et al. compared the decay curves of different sizes of NaYF_4_: 20%Yb,2%Er ranging from 15.3 nm to 27.2 nm, exhibiting the increase in the lifetime of both Er^3+^ and Yb^3+^ at 540 nm and 985 nm, respectively, as the nanoparticle dimension expanded [32].

#### 2.1.2. Inert Shell Passivation

The epitaxial shell could protect the excitation energy from trapping by surface ligands or solvent molecules, leading to a long-lived lifetime. Su et al. designed a core–shell–shell heterostructure to prolong the lifetime of upconversion emitters. An optically inert NaYF_4_ shell on the surface of NaGdF_4_@NaGdF_4_ core–shell nanoparticle could efficiently block the energy transfer from Gd^3+^ to surface quenchers, thereby promoting efficient upconversion emission and long lifetimes of Gd^3+^ [33]. Mao et al. successively compared the decay curves of NaYF_4_: Yb/Er/Mn (C_d_), NaYF_4_@NaYF_4_: Yb/Er/Mn (C/S_d_), NaYF_4_: Yb/Er/Mn@NaYF_4_ (C_d_/S) and NaYF_4_@NaYF_4_: Yb/Er/Mn@NaYF_4_ (C/S_d_/S), finding the lifetime became longer. Amongst them, the C/S_d_/S structure has two layers of NaYF_4_ interface contacting the active shell, which reduced surface defects and thus enhanced the upconversion process. Therefore, the lifetime can be prolonged by reducing energy defects and increasing the energy transfer rate [34].

CaF_2_ is a biocompatible and fluoride-rich matrix that is well used as a shell in upconversion nanomaterials, which can simultaneously suppress the interfacial diffusion of Ln^3+^ and surface quenching [35]. After heterogenous CaF_2_ layer was coated on the NaGdF_4_: Yb,Er, the lifetime of Er^3+^ at 532 nm prolonged from 89 μs to 162 μs, accompanied by the green (^2^H_11/2_, ^4^S_3/2_→^4^I_15/2_) and red (^4^I_15/2_→^4^I_15/2_) emissions of Er^3+^ enhanced 24.2 and 55.5 times, respectively [36]. When CaF_2_ grew on the core of NaYF_4_: 10%Yb,30% Nd, the lifetime of Yb^3+^ at 1000 nm increased from 51 μs up to 833 μs, indicating the surface lattice defects were minimized [37].

Interestingly, Su et al. provided an upconverted excitation lock-in (UCEL) mode to block the energy consumption of Gd^3+^ caused by lattice defects and impurities. NaYF_4_-based interlayer can effectively suppress the energy loss within the nanoparticles induced by inner lattice defects and promote energy recycling in the core domain. Therefore, the lifetime and the emission intensity of the heterogeneous structure significantly increased compared with the homogeneous counterparts (Figure 2b) [38].

Increasing shell thickness could suppress the non-radiative effect and cross-relaxation. Liu et al. synthesized NaYF_4_: Yb^3+^,Er^3+^@mNaYF_4_ (m = 0, 1, 2, 3, 4, 5) core–shell nanoparticles with different shell thicknesses (7.5 nm, 9 nm, 10 nm, 11 nm, 11.8 nm and 16.5 nm). The corresponding lifetimes of these nanoparticles were measured to be 10.10 μs, 11.57 μs, 15.78 μs, 19.77 μs, 23.51 μs and 26.52 μs due to the protection of shell [39]. Furthermore, increasing heterogenous shell thickness could minimize surface quenching. Coating a CaF_2_ shell with a range of thickness from 0 nm to 5.3 nm on the NaYbF_4_, the lifetime of Yb^3+^ of 980 nm increased from 33 μs to 2.18 ms, excited at 920 nm measured by an optical parametric oscillator (OPO) laser. Therein, when the CaF_2_ shell thickness reached 2.6 nm, the luminescence lifetime value was approximate to the radiative lifetime of Yb^3+^ [40].

#### 2.1.3. Active Shell

With regard to the active shell, Liu et al. proved that increasing the concentration of sensitizer in the shell had adverse impacts on the luminescence intensity and lifetime enhancement. In a NaYF_4_: 20%Yb,2%Er@NaYF_4_: m%Yb (m = 0, 10, 20) nanoparticle, the lifetime of Er^3+^ and Yb^3+^ at 540 nm and 985 nm decreased when the concentration of Yb^3+^ in the shell increased. This can be attributed to the fact that more sensitizers in the shell increased the possibility of energy trapped by the surface defect. In addition, a lower energy transfer efficiency was observed due to the longer distance between sensitizer and emitter compared with that in the counterpart consisting of inner sensitizers [32].

A longer migration distance imparted by thicker energy migration layers or an increased number of migration steps may prolong luminescence lifetime. In a core/multishell NaYF_4_@NaYbF_4_@NaYF_4_: Yb^3+^/Tm^3+^@NaYF_4_ nanoparticle, the thickness of the energy migration layer (NaYbF_4_) increased from 0, 1.5, 3.0, 5.0 to 8.0 nm gradually, and the lifetime at 808 nm increased from 867, 1027, 1162, 1201 to 1282 µs, respectively [41]. When the thickness of the shell doped with sensitizers (i.e., Yb^3+^) increased, the lifetime of activators was longer in the core due to Yb energy migration. Increasing the thickness of the second layer in the β-NaGdF_4_: 25%Yb,1%Tm@NaYF_4_: 10%Yb@ NaNdF_4_: 10%Yb@NaYF_4_ from 1.5 nm to 5.4 nm, the lifetime at 475 nm prolonged from 632 μs to 836 μs [42]. In addition, with NaGdF_4_@NaGdF_4_: Yb,Er@NaYF_4_: Yb@NaNdF_4_: Yb used as an experimental model, the lifetime at 1525 nm was enhanced when increasing the thickness of the energy relay layer (NaYF_4_: Yb), which was attributed to the enlarged distance from sensitizers to emitters. A similar trend was also observed in Ho^3+^-doped nanoparticles (Figure 2c). Meanwhile, a certain Yb^3+^-doped shell thickness corresponded to the determined capacity of Yb^3+^ sublattice. Therefore, increasing the concentration of emitters could accelerate the stored energy into luminescence emission, resulting in a shortened lifetime. As the proportion of Er^3+^ rose from 0.5% up to 32%, the lifetime at 540 nm decreased from 101 μs to 13 μs [43].

### 2.2. Changing Concentration of Sensitizer and Emitter

**Figure 3:**
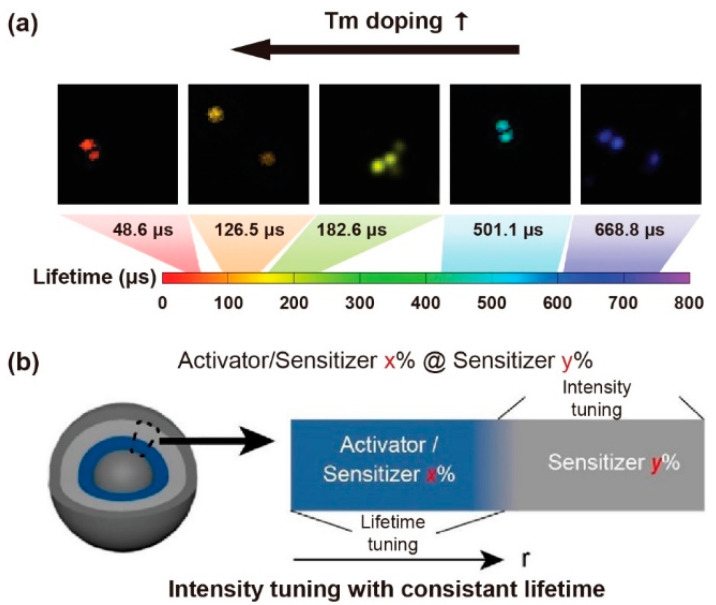
(**a**) The time-resolved confocal images of NaYF_4_: Yb,Tm nanoparticles and the lifetime tuning scheme by changing Tm^3+^ doping concentrations. Reproduced with permission from [20]. Copyright 2014, Nature Publishing Group. (**b**) Diagrammatic illustration of consistent lifetime with tunable intensity by doping various sensitive gradients in the shell. Reproduced with permission from [47]. Copyright 2021, WILEY-VCH.

Excessive emitters would increase the probability of cross-relaxation, leading to concentration quenching. For a Yb^3+^-Er^3+^-Ho^3+^ tri-doped nanoparticle, higher content of Ho^3+^ could shorten the interionic distances of Yb^3+^-Er^3+^/Ho^3+^, Er^3+^-Ho^3+^ and Ho^3+^-Ho^3+^, which could enhance the non-radiative process and energy transfer (ET) sensitization. A decrease in the lifetime of Ho^3+^ (643 nm, 750 nm, 895 nm), Er^3+^(525 nm, 545 nm, 655 nm, 810 nm, 845 nm) and Yb^3+^ (1020 nm) was observed when increasing the concentration of Ho^3+^ [44]. Similarly, as the mole content of Tm^3+^ rose from 0.2% to 8% in NaYF_4_: 20%Yb,x%Tm (40 nm), the lifetime of the blue emission was reduced from 662.4 µs to 25.6 µs (Figure 3a). Meanwhile, as the mole percentage of Yb^3+^ rose from 10% to 30% in NaYF_4_: x%Yb,1%Tm, the lifetime of the same blue emission decreased from 206.7 µs to 120.2 µs [20]. The higher concentration of part of emitters would likely cause self-aggregation and cross relaxation, resulting in a shortened lifetime. When the Er^3+^ concentration was varying from 1% to 70% in the β-NaYbF_4_@NaY_0.8−*x*_Er*_x_*Gd_0.2_F_4_@ NaY_0.8_Gd_0.2_F_4_, the lifetime of Er^3+^ at the level of ^4^S_3/2_ (540 nm) decreased sequentially. The shorter lifetime was attributed to the noneffective passivation on the surface when dopant with a higher concentration of emitters. Due to the greater energy from Yb^3+^ (^2^F_5/2_) being transferred to Er^3+^ (^4^S_3/2_, ^4^S_9/2_), the lifetime of Yb^3+^ was shortened. With the lack of cross-relaxation pathways for the ^4^S_9/2_ level of Er^3+^, the lifetime rarely has significant changes with the Er^3+^ concentration variation. Interestingly, the lifetime of Er^3+^ at 654 nm in the β-NaYbF_4_@NaY_0.8−*x*_Er*_x_*Gd_0.2_F_4_ structure had a long lifetime due to more incredible energy transferring to the surface [45].

Simultaneous excitation of two Yb^3+^ ions can produce Yb^3+^ dimers with higher excitation energy, which could upconvert photons to Tb^3+^. To study the composition-dependent emission lifetimes and the effect on the energy transfer efficiency, Yan et al. employed the Tb^3+^-Yb^3+^-Nd^3+^ co-doped NaGdF_4_: 80%Yb,10%Tb@NaGdF_4_: 50%Nd,10%Yb nanoparticles with varied doping concentrations as the study model. The lifetime of Tb^3+^ reached 1.76 ms when the content of Yb^3+^ rose from 20% to 80% because more Yb^3+^ facilitated the formation of Yb^3+^ dimer. The lifetime at 542 nm is slightly prolonged by 0.06 ms when the proportion of Tb^3+^ increased from 5% to 10% due to promoted energy transfer from Yb^3+^ dimer to Tb^3+^. In addition, when the content of Nd^3+^ increased from 10% to 50%, near-infrared absorption intensity improved, the lifetime of Tb^3+^ at 542 nm and Yb^3+^ at 1000 nm both increased, indicating more energy was transferred to Tb^3+^ and Yb^3+^ [46].

A constant lifetime can be obtained when the doping content of the sensitizer is changed. To investigate the relationship between lifetime decay behavior and luminescence emission intensity, core–shell structure of NaYF_4_@NaYF_4_: x%Yb,1%Tm@ NaYF_4_: y%Yb@NaYF_4_ nanoparticles was developed by Zhang et al. Changing the mole content of Yb^3+^ in the first shell from 20% up to 80%, the emissive intensity changed while the luminescence lifetime at 475 nm kept constant, suggesting a constant lifetime with different emissive intensity could be obtained. When the concentration of Yb^3+^ in the first and second shell layers was changed, the varied lifetime (1256 ms to 310 ms) was obtained with a constant emission intensity (Figure 3b) [47]. For the mentioned nanoparticles, the declined Yb^3+^ concentration increased the mean value of the distance between Yb^3+^ and Tm^3+^ ions, leading to a longer lifetime due to the weakened back energy transfer process. The Yb^3+^ concentration in the energy transfer upconversion layers was decreased from 99%, 70%, 50%, 40%, 20% to 10%, resulting in the lifetime at 808 nm being increased from 1282, 1315, 1481, 1618, 1721 to 2157 μs, respectively [41]. Besides, the Tm^3+^ could be served as sensitizers and transfer energy to Yb^3+^, Ho^3+^ and Er^3+^ when excited at 808 nm and 1208 nm, respectively. The lifetime of Yb^3+^ (980 nm), Ho^3+^ (1180 nm) and Er^3+^ (1525 nm) decreased with the increase of the Tm^3+^ molar ratio [48].

### 2.3. Adjusting the Energy Transfer Channel

**Figure 4:**
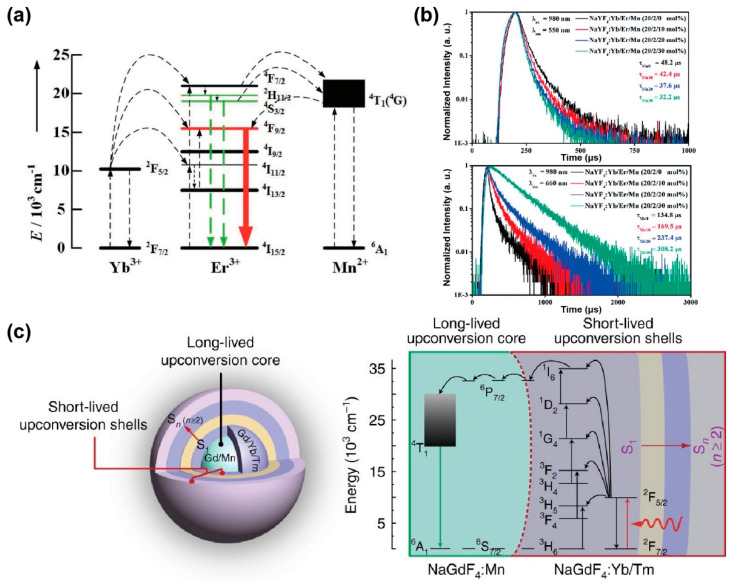
(**a**) The diagram for energy transfer mechanism among Yb^3+^, Er^3+^ and Mn^2+^ under 980 nm excitation. (**b**) Luminescence decay curves of Er^3+^ at 550 nm and 650 nm in NaYF_4_: Yb^3+^/Er^3+^ nanoparticles with different Mn^2+^ concentrations (0, 10, 20 and 30 mol%). Reproduced with permission from [34]. Copyright 2016, Royal Society of Chemistry. (**c**) Structural design of core–multishell nanoparticle and the energy transfer pathway among the Yb^3+^, Tm^3+^, Gd^3+^ and Mn^2+^ ions for the short- and long-lived upconversion luminescence under 980 nm excitation. Reproduced with permission from [51]. Copyright 2017, Nature Publishing Group.

The decay process of an excited state is inversely proportional to the energy transfer rate and the radiative and non-radiative transition rates. Tri-doped nanoparticles of NaYF_4_@NaYF_4_: Er^3+^/Yb^3+^/Mn^2+^@NaYF_4_ were synthesized by Mao et al., exhibiting the new energy transfer process between Mn^2+^ and Er^3+^. With a non-radiative energy transfer channel created between the level of Mn^2+^ (^4^T_1_) and Er^3+^ (^2^H_11/2_ and ^4^S_3/2_), the overall transition rate increased owing to the resonance energy transfer, leading to a reduced lifetime of Er^3+^ at 550 nm. The presence of Mn^2+^ ions promoted the relaxation of the ^4^S_3/2_ energy level, and the red emission of Er^3+^ increased with the shortening of the decay time. Therefore, the increased population density of Mn^2+^ caused the decreased radiative transition rate of Er^3+^ (^4^S_3/2_ and ^2^H_11/2_) turning down to ground state, resulting in an enhancement of red emission due to energy transfer from Mn^2+^ (^4^T_1_) to Er^3+^ (^4^F_9/2_) (Figure 4a). Meanwhile, the increased lifetime of Er^3+^ at 650 nm also verified the role of Mn^2+^ in energy transfer trace according to the decay curves of various content of Mn^2+^(0%, 10%, 20%, 30%) (Figure 4b) [34].

Introducing transition metal ions with a long lifetime into conventional UCNPs is particularly attractive. The lifetime of Mn^2+^ ions could be modulated by crystal-site engineering. Liu et al. tuned the luminescence properties of Mn^2+^ in core–multishell nanoparticles by doping Ca^2+^ or Mg^2+^, changing the output color from green to yellow and prolonging its lifetime [49]. The spin-forbidden transition of Mn^2+^ occurs between ^4^T_1_→^6^A_1_, allowing a longer fluorescence lifetime than lanthanide emitters. Because of the larger energy mismatch between Yb^3+^ and Mn^2+^, the Yb^3+^-Mn^2+^ dimer is difficult to form. However, Zhang et al. reported the successful preparation of Yb^3+^-Mn^2+^ dimers, obtaining a substantial long lifetime of Eu^3+^ (91 ms), while the normal UC lifetime of Eu^3+^ is only about 7 ms. In addition, there is a dynamic population balance between the energy state |^2^F_7/2_,^4^T_1_(4G)〉(Yb^3+^–Mn^2+^ dimers) and ^5^D_0_ (Eu^3+^), causing the sustained energy transferring from Yb^3+^–Mn^2+^ dimers to Eu^3+^ [50].

The long-lived Mn^2+^ integrated with the short-lived lanthanide particle platform could establish a new energy transfer pathway, and then affect the whole decay process. In a NaGdF_4_: 30%Mn@NaGdF_4_: 49%Yb,1%Tm@NaYF_4_ nanoparticle, Gd sublattice-mediated energy migration facilitates Mn^2+^ upconversion luminescence, leading to a decrease in the lifetime of Gd^3+^ at 311 nm (^6^P_7/2_→^8^S_7/2_) from 6.5 to 4 ms (Figure 4c) [51]. As a result, the lifetime of lanthanide ions may be affected when the external ions introduced and interfered with the energy transfer channels.

Doping various amounts of Gd^3+^ into the NaYF_4_ host nanocrystals could regulate the upconversion photoluminescence lifetimes. Xie et al. prolonged the lifetime of Er^3+^ (^4^S_3/2_→^4^I_15/2_, ^4^F_9/2_→^4^I_15/2_) by utilizing the Gd^3+^, substituting for Y^3+^ and Yb^3+^ in the crystal lattice of NaYF_4_ host, which attributed to the energy transfer rate decrease caused by the average sensitizer-activator separation increasing [52].

### 2.4. Fluorescence Resonance Energy Transfer

**Figure 5:**
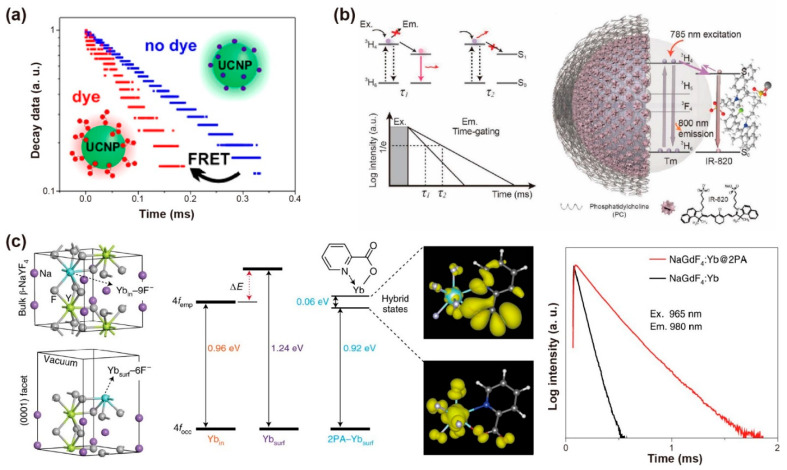
(**a**) Schematic diagram of the lifetime changing due to the existence of FRET process from UCNPs to dye molecules. Reproduced with permission from [54]. Copyright 2017, American Chemical Society. (**b**) Schematic representation of the structure of the NaYF_4_: Tm@PC-IR-820 nanocomposites, the absorbance and emission in the same transition (^3^H_6_–^3^H_4_) of NaYF_4_: Tm nanoparticles and the variation of lifetime affected by the amount of IR-820 dye molecules. Reproduced with permission from [55]. Copyright 2019, WILEY-VCH. (**c**) The optimized structure shows ytterbium atoms located in the interior (Yb_in_) and exterior (Yb_surf_) position, the simulated 4f energy levels of ytterbium atoms, the spatial distribution of the charge densities for coupling states and the lifetime decay curves of Yb^3+^ at 980 nm with and without 2PA capping on the NaGdF_4_: 5%Yb nanoparticles (10 nm) excited at 965 nm. Reproduced with permission from [59]. Copyright 2021, Nature Publishing Group.

An efficient energy transfer pathway could be established in fluorescence dye-loaded rare-earth nanocrystals, which enables luminescence lifetime tuning [53]. In contrast to conventional molecular donor–acceptor pairs, the energy transfer efficiency is related to the distances between lanthanide-doped nanoparticles, and thus significantly depends on the nanoparticle diameter. Hirsch et al. synthesized NaYF_4_: 20%Yb,2%Er with the precisely controlled size spanning from 10 to 43 nm, and coated with sulforhodamine B and rose bengal by ligand exchange. The nanoparticles with a mean diameter ranging from 20 to 25 nm possessed an optimum efficiency of 50–60%. The lifetime of Er^3+^ at 600 nm decreased primarily due to the competition of non-radiative surface deactivation at the smaller surface-to-volume ratios (Figure 5a) [54]. Li et al. loaded the IR-820 on NaYF_4_: Tm to construct a FRET system. Luminescence decay from ^3^H_6_→^3^H_4_ transition was used as a detection signal. When the energy accepter (IR-820) was attached to the donor (Tm^3+^: ^3^H_4_) under 785 nm excitation, the lifetime of Tm^3+^ at 800 nm decreased because of luminescence resonance energy transfer (Figure 5b) [55]. Su et al. loaded the IR-806 on the NaGdF_4_: 49%Yb,1%Tm@NaYF_4_: 20%Yb@NaGdF_4_: 50%Nd,10%Yb@NaGdF_4_ nanoparticles to improve the ultraviolet luminescence intensity. With the back energy transferred from the nanoparticles to dye molecules, the decreased lifetimes of Gd^3+^ and Tm^3+^ ions were observed at 253, 276, 290, 310, 360 and 475 nm [56]. An organic fluorescent dye as an antenna could be used to broaden and increase absorption for UCNPs, allowing the energy to flow to dye molecules. Meanwhile, the hybrid system between dye molecules and UCNPs creates a new energy diffusion pathway, increasing the radiative transition process. Li et al. added the Cy3-SO_3_ into a NaYF_4_: 20%Yb,2%Er@CaF_2_ solution to construct an energy dissipation channel, which could transfer energy to the dye by the radiative transition. As a result, the luminescence lifetime of Er^3+^ at 488 nm decreased with the concentration of Cy3-SO_3_ increase (0.67, 2, 4, and 5.33 μM). The reduced lifetime value verified the non-radiative energy transfer process between Er^3+^ and Cy3-SO_3_ [57].

The triplet excitons could be trapped by inter- or intra-molecular interactions and prolong organic phosphorescence. For example, Yb^3+^ luminescence could be generated by organic Yb^3+^ complexes and hybrid organic-conjugated Yb^3+^-doped nanoparticles. Ye et al. prepared a composite thin film, in which the Yb^3+^ ions are incorporated with tetrakis-(pentafluorophenyl)imidodiphosphinate to form the Yb(F-TPIP)_3_ chelate, while zinc salt of 2-(tetrafluoro-2-hydroxyphenyl)tetrafluorobenzothiazole (Zn(F-BTZ)_2_) served as the organic chromophore. The Zn(F-BTZ)_2_ possessed the emission ranging from 450 nm to 900 nm under 405 nm excitation, and gave rise of the Yb^3+^ emission centered at 975 nm from the transition of Yb^3+^: ^2^F_5/2_→^2^F_7/2_. Note that the intrinsic lifetime of Yb^3+^ at 1 μm is about ~1 ms. The lifetime of sensitized organic Yb^3+^ compounds could prolong up to ~0.3 s. The prolonged emission lifetime was demonstrated by dynamic equilibrium due to the energy transfer process from long-lived organic triplet excitons [58].

The surface ligand coordination could reconstruct the crystal-field splitting and orbital hybridization, and narrow the gap of the 4d orbitals between inner and surface lanthanide sensitizers. For example, after the bidentate picolinic acid (2PA) molecules coordinated to NaGdF_4_: Yb nanoparticles, a longer lifetime (289 μs) at 980 nm was observed. The results confirmed that 2PA molecules could activate the dark surface layers and facilitate energy migration in the Yb^3+^ sublattices. The Yb^3+^-2PA coupling facilitated energy migration by 4f-orbital energy resonance within the ytterbium sublattice, which can reduce surface defects to hinder energy diffusion. Density functional theory (DFT) verified that 2PA coating could narrow the gap between the superficial and inner Yb^3+^ by lowering the empty 4f levels. Rigid ligands also stabilized the excited state of superficial Yb^3+^, prevented the superficial lanthanide ions from the solvent and fluoride vacancy-induced quenching, and thus significantly suppressing multiphonon non-radiative decay (Figure 5c) [59].

### 2.5. Changing Temperature

**Figure 6:**
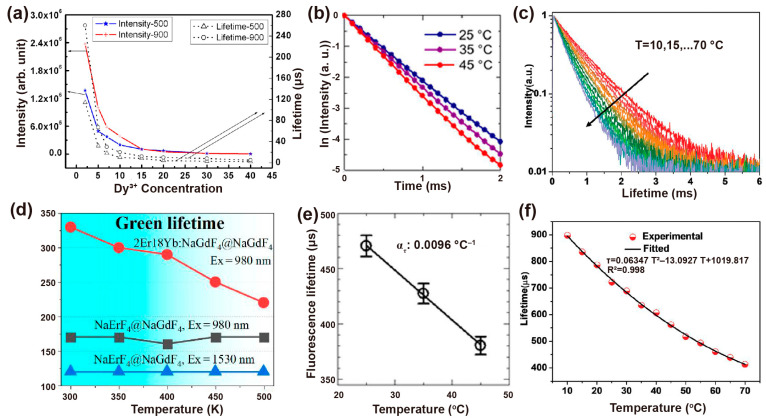
(**a**) Intensity (left) and the corresponding decay lifetime of Dy^3+^ at ^4^F_9/2_ states (right) with the dependent variable of Dy^3+^ concentration when excited at 310 nm. Reproduced with permission from [60]. Copyright 2009, AIP Publishing. (**b**) Luminescence decay curves of NaYF_4_: Nd^3+^,Yb^3+^ at different experimental temperatures and (**e**) the corresponding calibration curve of temperature vs. luminescence lifetime. Reproduced with permission from [63]. Copyright 2019, Nature Publishing Group. (**c**) The decay curves of the measured NaYF_4_@NaYF_4_: 20%Yb^3+^,60%Nd^3+^@CaF_2_ nanoprobe at various temperatures and (**f**) corresponding nonlinear fitted curves between measured lifetime and temperature ranging from 10 °C to 70 °C. Reproduced with permission from [66]. Copyright 2020, WILEY-VCH. (**d**) Negative correlation curves of the lifetime of Er^3+^ at ^4^S_3/2_ versus ambient temperature for NaErF_4_@NaGdF_4_ and NaErF_4_: 18%Yb,2%Er@NaGdF_4_ nanoparticles. Reproduced with permission from [67]. Copyright 2020, MDPI.

The synthesized process would affect the crystallinity, phase state and volume of nanoparticles and thus influence the decay behaviors. Vatsa et al. studied the decay process of GdVO_4_: Dy^3+^ nanoparticles after heat treatment. When heated from 500 °C to 900 °C, the lifetime of Dy^3+^ (^4^F_9/2_ level) extended from 114 μs to 260 μs due to the reduction of the non-radiative process by surface inhomogeneities. This increase in lifetime can also be ascribed to the decrease in the surface defects with the particle size increases in the heat treatment process (Figure 6a) [60]. For YVO_4_: Ln^3+^ (Ln^3+^ = Dy^3+^, Eu^3+^) nanoparticles, the increase in covalent bond interaction caused by heat treatment led to a red shift in V–O charge transfer (CT). Similarly, the lifetimes of Dy^3+^ at ^4^F_9/2_ and Eu^3+^ at ^5^D_0_ increase with temperature from 500 °C to 900 °C due to the reduction of the non-radiative process on the surface of the particles [61].

As is well known, the decay time constant is inversely proportional to the radiative and non-radiative transition rates in the cross-relaxation process. The luminescence lifetime decreases with the increase of ambient temperature in most cases. The decay time with specific emissions produced by radiative transition rarely varies with temperature, while the non-radiative decay rate changes significantly with temperature [62]. For example, the lifetime of Yb^3+^ at 1000 nm reduced from 470 ± 11 μs to 390 ± 12 μs in NaYF_4_: Nd^3+^, Yb^3+^ nanoparticles as the temperature rose from 25 °C to 45 °C. While the thermal coefficient α_τ_ calculated by the TGI system was almost unchanged (−0.0092~−0.010 °C^−1^) (Figure 6b,e) [63]. Moreover, cross-relaxation between Tm^3+^ (^1^G_4_) usually occurs when raising the emitter concentration or temperature. Yu et al. compared the sensitivity of β-PbF_2_: Tm^3+^/Yb^3+^ with different Tm^3+^ doping concentrations. They found that the relative sensitivity maximum values of ^1^G_4_ state lifetime in 0.0005Tm, 0.01Tm and 0.05Tm nanoparticles are 0.16%K^−1^, 0.26%K^−1^ and 0.46%K^−1^ at 488K, respectively, indicating the potential ability as an indicator of upconversion luminescence lifetime-based thermometer [64].

In addition, the host matrix has a significant effect on thermal sensitivity. Díaz et al. found that oxide materials are more sensitive than fluoride ones by comparing the decay curves of NaYF_4_: Er,Yb and NaY_2_F_5_O: Er,Yb nanoparticles at room temperature and 60 °C. [65]. The temperature dependency of Yb^3+^ emission lifetime in NaYF_4_@NaYF_4_: Yb^3+^,Nd^3+^@CaF_2_ nanoparticles was determined by the energy transfer and back energy transfer rate, the energy migration process (among Yb^3+^), as well as radiative and non-radiative transition. Both the concentration of Nd^3+^ and Yb^3+^ affected the temperature sensitivity by changing the distance of Yb^3+^-Yb^3+^ and Yb^3+^-Nd^3+^, which in turn affected the back energy transfer processes from Yb^3+^ to Nd^3+^ and energy migration between Yb^3+^ ions. NaYF_4_@NaYF_4_: 20%Yb^3+^,60%Nd^3+^@CaF_2_ as the nanoprobe possessed optimum thermal sensitivity through varying doping concentrations of Yb^3+^ and Nd^3+^, in which the lifetime of Yb^3+^ at 980 nm descended from 898 μs to 450 μs when the temperature increased from 10 °C to 64 °C. (Figure 6c,f) [66].

Interestingly, Li et al. demonstrate the lifetime compensation with temperature in NaErF_4_@NaGdF_4_ core–shell nanoparticles. The temperature-independent lifetime is attributed to the balance between lattice expansion (prolonging the lifetime) and thermal quenching (shortening the lifetime). A considerable energy migration process occurs in the high-doping concentration of Er^3+^, and the efficiency is proportional inversely to the average donor-acceptor distance with sixth order of magnitude. As a consequence, elevated temperature induces the lattice to expand, leading to a longer transfer distance, and ultimately prolonging the lifetime of Er^3+^. However, the prolonged lifetime caused by lattice expansion compensated for the difference value of the shorter lifetime aroused by thermal quenching, resulting in the temperature-independent lifetime (Figure 6d) [67].

## 3. Bioapplications

With proper surface functionalization, lanthanide-doped nanoparticles possessed good biocompatibility and low toxicity [68]. Additionally, the pulsed laser illumination has a lower thermal effect than continuous-wave laser emission, which was harmful in biological applications [23]. Therefore, lanthanide-doped nanoprobes combined with lifetime sensing technology involving time-gated and lifetime coded imaging is widely used in biology and medicine [18]. This review gives a perspective of lifetime-based sensing and imaging in biological applications at molecule, cell, organ and living body levels.

### 3.1. Ions and Molecules Detection

**Figure 7:**
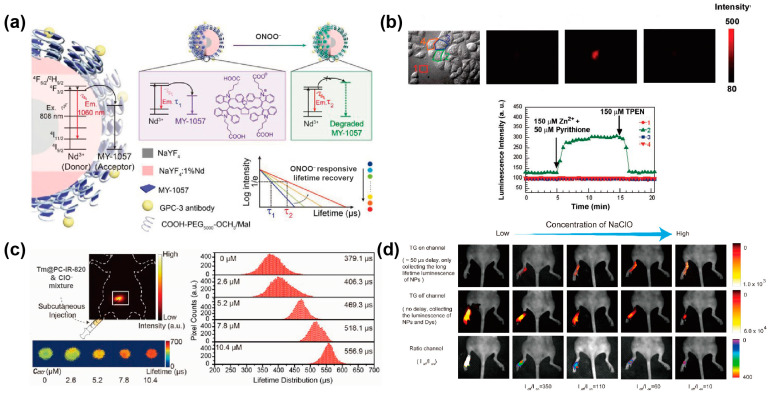
(**a**) Schematic diagram of nanosensor DSNP@MY-1057-GPC responding to ONOO^−^. Reproduced with permission from [69]. Copyright 2020, WILEY-VCH. (**b**) Time-resolved luminescence imaging of Zn^2+^ in living HeLa cells. Reproduced with permission from [71]. Copyright 2007, American Chemical Society. (**c**) Simulated luminescence lifetime imaging of the detection of responsive ClO^−^. Reproduced with permission from [55]. Copyright 2019, WILEY-VCH. (**d**) The images of living mice in vivo with injection of 25 μL sodium hypochlorite solution and 25 μL NaYF_4_: Nd@Cy860@PC aqueous solution (3 mol/L). Reproduced with permission from [72]. Copyright 2019, American Chemical Society.

The quantitative detection of targets based on intensity was unreliable due to the inhomogeneous scattering and absorption. Utilizing the lifetime variation of donor and acceptor, lifetime sensing based on the FRET mechanism could monitor ions’ concentration accurately. Zhang et al. integrated the Nd^3+^-doped nanoparticle (energy donor) and MY-1057 (energy acceptor) to detect peroxynitrite (ONOO^−^) in the tumor-microenvironment based on the lifetime of NIR-II emission. The luminescence lifetime of the nanosensor at 1060 nm shortened with the increase in the amount of the surface dye molecules. The energy acceptor MY-1057 was destructed after reacting with reactive nitrogen species (especially ONOO^−^), resulting in the lifetime being recovered. Furthermore, the lifetime of the nanosensor would recover from 202 μs to 303 μs continuously after ONOO^−^ addition, showing linearity corresponding to ONOO^−^ concentration while independent of the penetration depth (0, 2, 5 mm). As a result, the ONOO^−^ concentration could be measured under unknown tissue penetration depth on a basis of standard curve due to the reliable lifetime-based ONOO^−^ detection (Figure 7a) [69]. Besides, Li et al. synthesized a lifetime-responsive nanocomposite consisting of NaYF_4_: Tm nanoparticles and IR-820 dye molecules. The energy transfer from Tm^3+^-doped nanocrystal to IR-820 provided a tunable luminescence lifetime. ClO^–^ can destruct the dye and recover the lifetime of Tm^3+^. Based on lifetime changes, the concentration of ClO^–^ could be detected (Figure 7c) [55].

Time-resolved fluorescence microscopy could monitor target fluorescence by distinguishing the differences of fluorescence lifetimes in the nanosecond regime. For metal ions, Zhang et al. utilized the time-resolved fluorescence signals of BSA/Tb^3+^ to detect metal ions, involving Cu^2+^, Co^2+^, Zn^2+^, Mn^2+^, Ni^2+^, Pb^2+^, Ag^+^, Li^+^, Na^+^, Fe^3+^, Ca^2+^, Mg^2+^, Al^3+^, K^+^, Cd^2+^, Cr^3+^ and Hg^2+^ in two pH buffers (7.4 and 8.5). A 2000 μs gate time and a 50 μs delay time were settled for time-resolved fluorescence spectra by recording at 548 nm. Furthermore, the sensing platform could distinguish the various concentrations of the identical metal ions and the variety of metal ions mixture, even in biofluids [70]. Nagano et al. designed luminescent Eu^3+^ complexes (Eu-7) for time-resolved, long-lived luminescence microscopy (TRLLM). With Eu-7 injecting into a single Hela cell, the lifetime window centered at 617 ± 37 nm of Eu^3+^-based luminescence was collected under 360 ± 40 nm excitation. An increased luminescence enhancement was observed when the intracellular Zn^2+^ mixed with pyrithione, and decreased with the addition of cell-membrane-permeable chelator TPEN (N,N,N’,N’-tetrakis(2-picolyl)ethylenediamine). As the delay times prior to counting and gate time were 70 and 808 μs, respectively, the intracellular Zn^2+^ concentration variation in living cells could be examined by using the TRLLM system with Zn^2+^-sensitive luminescent lanthanide probe [Eu-7] (Figure 7b) [71].

Li et al. designed a water-soluble nanocomposite NaYF_4_: 5%Nd and dye Cy860 enveloped by phosphatidylcholine (NPs@dye@PC). The Cy860 dye can quench the luminescence of lanthanide-doped nanoparticles. After hypochlorous acid (HCIO) reacted with organic dye molecules, the quenching process was broken, and the luminescence of lanthanide-doped nanoparticles was recovered. Meanwhile, the lifetime of Nd^3+^ at 893 nm was shortened from 51 μs to 16 μs due to the FRET and inner filter effect. Utilizing the time-gated technique and signal collection method, they used NaYF_4_: Nd@Cy860@PC nanoprobe to detect the concentration of HCIO in a living mouse model. Of note, the average relative deviations of HCIO concentration were only 0.61% and 1.74% via ratiometric detection, while the tissue depth increased up to 2 and 3 mm, respectively (Figure 7d) [72]. Yuan et al. designed and synthesized a time-gated luminescence TGL probe (TR-HCIO) for specific detection of HCIO, in which luminescent Tb^3+^ (energy donor) nanoparticles were conjugated with a rhodamine derivative (energy acceptor). After reacting with HCIO, the rhodamine emission at 580 nm increased while the Tb^3+^ emission at 540 nm decreased, resulting in an increase in the TGL intensity proportion of rhodamine to Tb^3+^ (I_560_/I_540_) up to ~9-fold. The luminescence lifetime of Tb^3+^ decreased from 588 μs to 254 μs due to the FRET process and has excellent linearity of the variation of HCIO concentration from 0.5 μM to 30 μM (r = 0.99). With dual signal outputting by ratiometric TGL and luminescence lifetime, TR- HCIO was applied to determine HCIO in HepG2 cells [73].

Vitamin C distribution and dynamic activities could be monitored by the time-gated luminescence microscopy. Yuan et al. developed a probe responding to ascorbic acid (vitamin C) conjugating two nitroxide radicals and a luminescent europium complex. The nitroxide radicals prevented the probes from emitting luminescence until vitamin C was added to form a hydroxylamine derivative. The probes responded to vitamin C concentration linearly with a limit of detection (LOD) of 9.1 nM, which is lower than electrochemical methods by two orders of magnitude. The method of time-gated luminescence microscopy enabled real-time and specific monitoring of the cellular uptake, endogenous production and mapping of vitamin C in living Daphnia magna with free background [74].

### 3.2. DNA Detection

**Figure 8:**
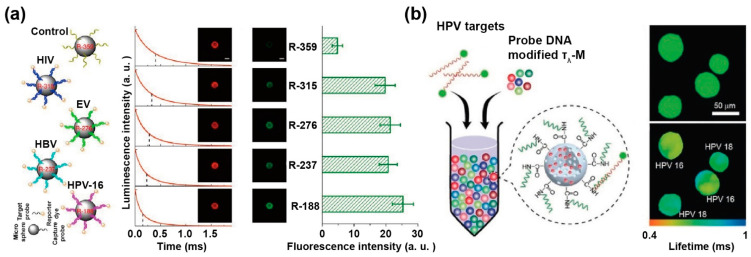
(**a**) Diagram of the five types of Eu-LRET microspheres conjugated to five different DNA sequences, in which the microspheres and amounts of pathogen DNAs were confirmed by the luminescence decay curves of Eu^3+^ and reporter fluorescence intensities, respectively. Reproduced with permission from [75]. Copyright 2014, Nature Publishing Group. (**b**) Schematic diagram of τ_λ_-M for detecting HPV subtypes, and the confocal images with the corresponding lifetime images after incubating complementary target DNAs, respectively. Reproduced with permission from [42]. Copyright 2018, WILEY-VCH.

The pathogen DNA strands could be detected by using lifetime coding and decoding with downconversion lanthanide luminescence in microspheres. Jin et al. encapsulated a trivalent europium complex of thenoyltrifluoroacetonate Eu (donor) and a hexafluorophosphate salt of cationic coumarin 50 (acceptor dyes) into porous polystyrene beads by solvent swelling. The average donor-to-acceptor distance could be manipulated by stepwise varying their concentrations, achieving the fine-tuned lifetimes of the microspheres. They carried out a biological experiment for high-throughput simultaneous detection of different pathogen DNAs (single strands), including human immunodeficiency virus, Ebola virus, hepatitis B virus and human papillomavirus (HPV) 16. The pathogen DNAs were added into the test panel composed of five types of conjugated microspheres encoded with different life spans, and Qdot 565 was added as a reporter dye. With the time-resolved orthogonal scanning automated microscopy analysis, the lifetime of Eu^3+^ complex in lanthanide-encoded microspheres were recovered by detecting the time-gated luminescence, which can identify their types of pathogen DNAs by decoding the lifetimes (Figure 8a) [75].

Similarly, Zhang et al. synthesized a series of nanoparticles with a settled lifetime by regulating the doped proportion of activators and the thickness of the energy migration layer. These nanoparticles were loaded into microspheres and modified by the DNA probe with nine high-risk HPV subtypes. With fluorescence dye 6-carboxyfluorescein as the reporter for DNA detection, the nanoparticles modified with DNA probe were added into a solution including PCR amplified DNAs of HPV targets. The HPV positive sample both of HPV 16 and HPV 18 could be distinguished by using the time-resolved imaging scanning system (Figure 8b) [42].

### 3.3. Protein Detection

A strategy to detect protease is to utilize lanthanide nanoprobes, which exhibit the long lifetimes and strong luminescence in the presence of an amino group, and a reverse trend with the protection of acyl groups. Kikuchi et al. synthesized lanthanide-based antenna-chelator conjugates with aniline derivative groups as the antennas. This conjugation has strong luminescence and a long lifetime (1.5 ms) at 350 nm when calpain I and leucine aminopeptidase (LAP) are added. These probes could exclude background fluorescence signals, suitable for detecting protease activities such as LAP and calpain I by time-resolved assays [76].

**Figure 9:**
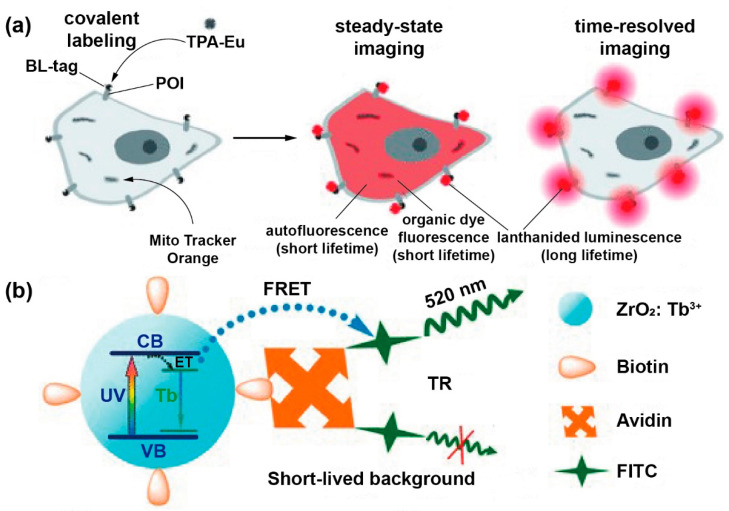
(**a**) Schematic representation of mutant β-lactamase-based protein labeling system, in which the luminescent lanthanide probe (TPA-Eu) was used for imaging cell-surface proteins (POIs). Reproduced with permission from [77]. Copyright 2011, WILEY-VCH. (**b**) Schematic diagram of TR-FRET detecting of avidin by employing biotinylated NCs (donor) and FITC (acceptor). Reproduced with permission from [79]. Copyright 2011, WILEY-VCH.

Similarly, Kikuchi et al. prepared a luminescent lanthanide probe TPA-Eu using time-resolved luminescence microscopy for protein imaging. The lifetime was 1.25 ms at 616 nm, which could be applied to detect TPA-Eu labeled on cell-surface proteins. The long lifetime could effectively separate the live-cell imaging from background signals (Figure 9a) [77]. Vuojola et al. constructed a hybrid FRET system consisting of Tb^3+^ with lanthanide-binding peptide (LBP) and green fluorescent protein (GFP). LBP can prolong the emission lifetime and can be applied in time-gated detection. After LBP and GFP were digested by enzyme, the long-lived sensitized acceptors were removed. The variation of the time-gated signals of terbium at 545 nm and the sensitized acceptor emission at 520 nm were monitored and used to detect the presence of caspase-3 inhibitor Z-DEVD-FMK [78].

The time-resolved detection could trace the biomolecules such as avidin with FRET biosensor, which can eliminate the background signals and improve the sensitivity. Chen et al. designed a hybrid system; NaYF_4_: Ce/Tb nanocrystals could transfer the energy to fluorescein isothiocyanate (FITC). The lifetime of Tb^3+^ at ^5^D_4_ decreased with the concentration of avidin improving from 0 to 500 nM. Using the model system, they could achieve an LOD of 5 nM (Figure 9b) [79]. By utilizing time-resolved fluorescence resonance energy transfer (TR-FRET), the avidin could be detected with the detection limit of 3.0 nM by using ZrO_2_ NP bioprobes [80]. In addition, Chen et al. synthesized CaF_2_: Ln^3+^ (Ln = Ce, Tb; Yb, Er; Yb, Tm) NPs with a size of sub-10 nm, which could sensitively detect avidin in homogeneous TR-FRET bioassays. Due to great spectral overlap, amino terminal fragment (ATF) coupled with CaF_2_: Ce, Tb NP (emitting at 491 nm) labeled FITC (emitting at 490 nm) for TR-FRET detection. Therefore, the excitation energy of CaF_2_: Ln^3+^ could be transferred to nearby FITC on account of the specific bond between avidin and biotin. The TR-FRET signal was enhanced gradually at the expense of the Tb^3+^ signal as the amount of avidin increased. The calibration curve for avidin concentrations from 0.1 nM to 430 nM exhibits that the signal of FITC/Tb in TR-FRET increases with the avidin concentration. The LOD is approximately 164 pM, which is the lowest detection limit for bioprobes on the basis of Ln^3+^-doped inorganic nanoparticles. Specially, they designed a system to detect soluble urokinase plasminogen activator receptor with ATF-coupled nanoparticles as the probes, whose LOD of tumor marker suPAR is 328 pM [81].

On the basis of the FRET mechanism, lanthanide nanoparticles can provide sensitive detection by using their decay time signals. Zhang et al. designed a hybrid system of UCNP-aptamer/ssDNA-pyropheophorbide-a (PPA)-doxorubicin (DOX) [UAS-PD] with 540 nm and 655 nm emission for targeted cancer therapy. The Black Hole Quencher-1 (BHQ-1) bore on ssDNA without the target cell. It could quench the luminescence of UCNPs at 540 nm due to FRET, but had a limited effect on 655 nm emission. A spontaneous conformational reorganization would occur when approaching specific cancer cells, moving ssDNA away from UCNPs and making PPA close to UCNPs. PPA decreased the luminescence intensity at 655 nm and recovered 540 nm emission due to FRET. Meanwhile, it affected the lifetime of both 540 nm and 655 nm. As a result, an extracellular cancer-specific biomarker PTK7 was developed to activate the UAS-PD as a probe. The lifetime of UAS-PD at 540 nm and 655 nm was measured to be 339.22 μs and 668.61 μs, respectively. Nevertheless, in the presence of PKT7, the lifetime of hybrid system increased by 476.33 μs at 540 nm, while the decay times at 655 nm decreased to 404.52 μs due to FRET from UAS-PD to BHQ-1 and PPA. Specially, the ratiometric lifetime signal provided an extremely low LOD of 3.9 nM for PTK7 [82].

The sensitivity of immunoassays could be improved by using lanthanide-labeled nanoparticles with time-resolved immunofluorometric assays (TrIFA). Li et al. coated the luminescent Eu^3+^ and Tb^3+^ chelates covalently on the surface of silica nanoparticles to conjugate the antibodies or bind antibodies. As a comparison, the lifetime was no longer than 0.3 ms for Eu^3+^-BHHCT after encapsulation in silica nanoparticles, while the lifetime of Tb^3+^-BPTA chelates decreased prominently from 2.68 ms to 1.52 ms after encapsulation. The as-prepared conjugates in TrIFA could detect hepatitis B e antigen (HBeAg) and hepatitis B surface antigen (HBsAg). Utilizing the conjugates, the TrIFA for HBsAg possessed a comparable or lower LOD (0.0092 μg/L) than ELISA while the TrIFA for HBeAg possessed a much lower LOD (10.0 National Centre Unit (NCU)/L) than ELISA. By synchronizing TrIFA, the detection limits reached 0.033 μg/L for HBsAg and 27.0 NCU/L for HBeAg, which is close to those of the individual assays [83].

### 3.4. Cell Labeling

**Figure 10:**
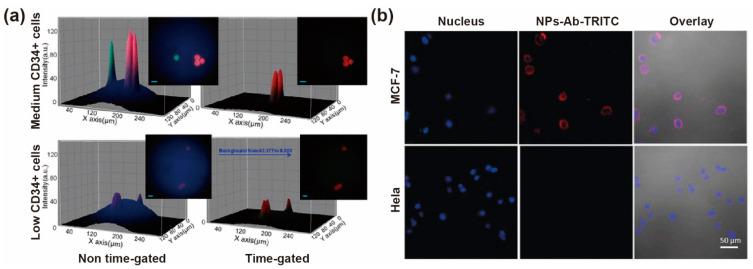
(**a**) The comparison of conventional UV fluorescence microscopy imaging (left) and time-gated luminescence microscopy imaging (right) of medium. Reproduced with permission from [84]. Copyright 2012, American Chemical Society. (**b**) Confocal laser scanning microscopy images of EpCAM-positive MCF-7 and EpCAM-negative HeLa cells incubated with NaEuF4–Ab–TRITC for 1.5 h. Reproduced with permission from [85]. Copyright 2019, WILEY-VCH.

Lanthanide bioprobes with time-gated detection enable rapid identification and quantification of cells bearing low-abundance surface biomarkers. CD34^+^ cells are the hematopoietic stem cells, whose surface possesses the specific expression of CD34 protein. Jin et al. synthesized functionalized polystyrene nanoparticles containing europium and stained CD34^+^ cells with a streptavidin–europium complex conjugate. Biotinylated anti-CD34 antibodies stained CD34^+^ cells, and were imaged by time-gated luminescence, suppressing autofluorescence background signal (Figure 10a). As a result, the signal intensity was improved by a factor of ~20, which could quantify the surface antigens of low expression on a single cell. Furthermore, with the assistance of an orthogonal scanning automated microscopy, they obtained the quantitative statistical data of numerous CD4 cells on microscopy slides. They separated 98% target cell population from stained cells with a coefficient of variation of 31% [84].

Precise detection of tumor cells is critical for diagnosing early-stage cancer, forcing researchers to develop highly sensitive methods. The lifetime-resolved luminescent lanthanide technology could obtain high signal intensity by suppressing the background fluorescence. Chen et al. synthesized anti-EpCAM-antibody-modified NaEuF_4_ NPs (NaEuF_4_–Ab) to detect circulating tumor cells (CTCs) in whole blood samples without CTC enrichment. This amplified the signal through enhanced dissolution of time-resolved photoluminescence and elimination of short-lived autofluorescence interference. The detection of blood breast cells had a LOD of 1 cell/well in a 96-well plate. As a result, the direct detection of blood breast cancer cells has a detection rate of 93.9% (14/15 patients) in cancer patients. The time-resolved photoluminescence could improve the signal to noise ratio of the confocal laser scanning microscope (CLSM). It amplified the signal through the enhanced dissolution of time-resolved photoluminescence and elimination of short-lived autofluorescence interference. Comparing CLSM images of EpCAM-positive MCF-7 and EpCAM-negative HeLa cells incubated with NaEuF_4_–Ab–TRITC for 1.5 h, the surface of MCF-7 cells emitted intense red luminescence but the surface of HeLa cells did not (Figure 10b) [85].

To increase the luminescent sensitivity, an effective strategy is to attach lanthanide chelates onto carrier molecules such as antibodies, which then were used to label surface antigens on cancer cells. Packer et al. developed a tetradentate β-diketonate-europium chelate for the immunodetection of prostate cancer cells (DU145). They conjugated MIL38 antibody to the chelate directly via lysine residues, and labeled a europium chelated secondary polyclonal antibody. As the DU145 cells (a prostate cancer cell line) were stained by conjugates, the time-gated luminescence microscopy was used to capture the images of immune-stained cancer cells while the cellular autofluorescence background was suppressed [86].

Another efficient method is to construct multiple lifetime channels, in which the lifetime could be precisely adjusted and detected. Zhang acquired a battery of Er-doped nanoparticles with settled lifetime at 1525 nm by control the thickness of energy relay layers and the concentration of activators. Three types of nanoparticles with settled lifetime were conjugated with primary antibodies to label the MCF-7 and BT-474 breast cancer cells, on a basis of detecting the biomarkers of breast cancer including progesterone receptor (PR), oestrogen receptor (ER) and human epidermal growth factor receptor-2 (HER2). Due to the different expression patterns of the three biomarkers for the two tumor subtypes, the biomarker expressions of the tumor subtypes could be quantified by analyzing the three lifetime composites according to a pattern recognition algorithm. The highest expression in the MCF-7 tumors came from ER (62.3%), followed by PR (17.9%) and HER2 (19.8%). On the other hand, the BT-474 tumors expressed a large amount of HER2 (46.6%) but moderate levels of PR (28%) and ER (25.4%), which were in accordance with the standard ex vivo immunohistochemistry assays [43].

### 3.5. Organ Imaging

**Figure 11:**
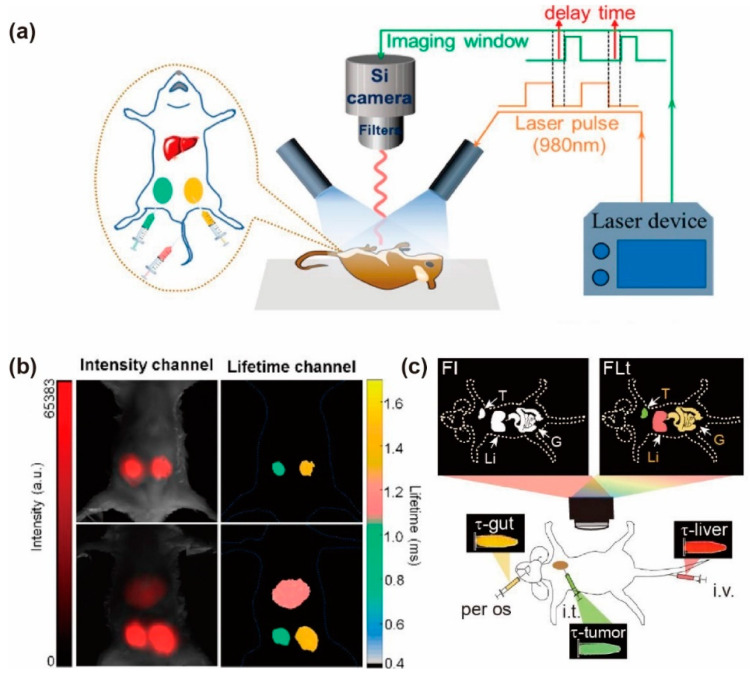
(**a**) Schematic diagram of nanoparticles with determined lifetime injecting into mouse, and then recorded by the home-built time-resolved luminescence imaging system. (**b**) The luminescence intensity (left) and lifetime (right) imaging of PAA-coated nanoparticles with two distinctive lifetimes injecting into a Kunming mouse and also into a second Kunming mouse (bottom). Reproduced with permission from [41]. Copyright 2020, American Chemical Society. (**c**) Schematic illustration of non-invasive NIR-II luminescence intensity and lifetime-based imaging for three diverse administration monitoring. Reproduced with permission from [87]. Copyright 2021, WILEY-VCH.

The practical use of UCNPs is still hampered by relatively shallow penetration depth. Comfortingly, lanthanide lifetime measurements are independent of tissue thickness, which can overcome the problem by lifetime coded technology. Chen et al. coated polyacrylic acid on a series of NaYF_4_@NaYbF_4_@NaYF_4_: Yb^3+^/Tm^3+^@NaYF_4_ via ligand exchange. They injected PAA-coated UCNPs (100 μL, 30 mg/mL) with the lifetime of 1158 μs into a Kunming mouse by tail vein injection, 20 μL of PAA-coated UCNPs (30 mg/mL) with lifetimes of 1528 μs and 920 μs into the right and left of the abdomen through subcutaneous injection, respectively. The time-delayed images were analyzed algorithmically by MATB. By taking advantage of temporal optical multiplexed upconversion with distinct lifetime-hued colors, liver and two abdomen subcutis could be seen clearly. The two close lifetimes could also be differentiated in in vivo imaging, indicating high temporal resolution abilities of the imaging system (Figure 11a,b) [41].

NIR-II luminescence has the advantage of reducing optical scattering. Er^3+^ emission at 1532 nm combined with lifetime sensing technology attracted specific attention. The fluorescent nanoprobes with Er^3+^ dopant in double interfaces (NaYF_4_@NaErF_4_: Ce@NaYbF_4_@NaErF_4_: Ce@NaYF_4_) was designed by Zhang et al. to generate strong luminescence intensity and regulate the lifetime distinguishably. The nanoparticles decorated with phospholipid were administrated via oral, intertumoral and intravenous injection into mouse baring with a subcutaneous tumor. Compared with other nanoparticles having larger luminescence intensity differences, these nanoprobes offered more accurate lifetime decoding for metabolically enriched organ imaging. During the 6 h monitoring period, it exhibited consistent characteristic lifetime compared with the agents in gut and tumor (Figure 11c) [87].

### 3.6. Thermal Sensing

Utilizing the temperature-dependent lifetime of lanthanide nanomaterials, lifetime-based thermometers have been developed to detect the temperature in the biologic microenvironment. Férid et al. synthesized La_2_O_3_: Tm,Er,Yb UCNPs and used it as luminescent thermal probes to detect the temperature in the simulative biological tissue. The employed fluorescence lifetime is from the ^1^G_4_→^3^H_6_ transition of Tm^3+^ at 480 nm. UCNPs were placed at the same point on a biological tissue (0.15 mm thickness), and then were focalized by a 1450 nm heating diode laser and excited by a 980 nm pulsed laser. The fluorescence lifetime at 480 nm was measured with heating laser power varying from 30 mW to 130 mW. Hence, the sub-tissue temperature could be calculated by the achieved decay curves [88]. In an ex vivo experiment, a probe of water-dispersed NaY_2_F_5_O: Er,Yb nanoparticles was injected into a chicken breast at 1 mm depth and heated by a laser beam. The luminescence decay curves excited by a 980 nm diode laser were recorded by a double beam confocal microscope. According to the calibration curves, the sub-tissue temperature could be determined by the level of a shortened lifetime [65].

**Figure 12:**
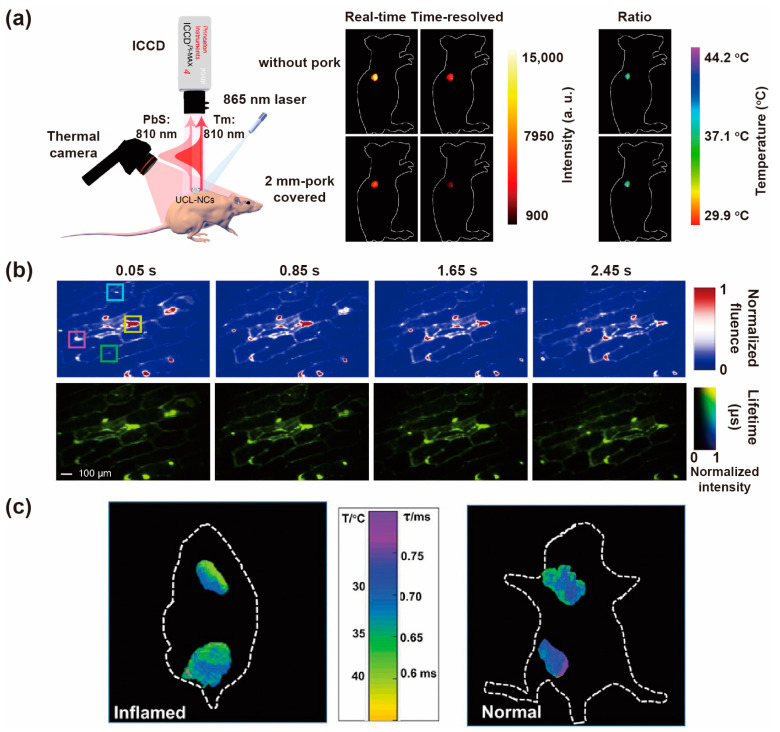
(**a**) Diagram of surficial and intratumoral detection in vivo and corresponding real-time, time-resolved and ratio UCL imaging without and with a 2 mm-pork slice covering under 865 nm excitation. Reproduced with permission from [89]. Copyright 2020, Nature Publishing Group. (**b**) The time-integrated images of a dynamic onion epidermis cell sample with labeling UCNPs. Reproduced with permission from [90]. Copyright 2021, Nature Publishing Group. (**c**) Thermographic lifetime imaging of the nanosensors in the inflamed and normal mouse, showing the precise position of temperature distribution. Reproduced with permission from [66]. Copyright 2020, WILEY-VCH.

Integrating with QDs, the lanthanide upconversion nanomaterials exhibited excellent temperature sensing capability by utilizing arithmetic methods. Li et al. fabricated the hybrid upconversion nanoclusters (UCL-NCs) containing PbS QDs and NaYbF_4_: 0.5%Tm@NaYF_4_: 10%Yb@NaYF_4_: 50%Nd (Tm-UCNPs) through an evaporation-induced self-assembly method. Both PbS QDs and Tm-UCNPs emitted around 810 nm through the upconversion process under 865 nm excitation. The lifetime of PbS QDs is responsive to the temperature at ns level, while the lifetime of Tm-UCNPs is fairly long, reaching a magnitude of μs, and is independent of temperature. Pork tissue with different thicknesses mimics biological tissues to study the temperature variation of UCL-NCs. *I*_Sum_ was obtained by the real-time imaging, and the *I*_Tm_ was acquired by a 20 μs delayed time-resolved spectrometer. The experimental data was linearly fitted according to the ratio obtained from different thicknesses according to the formula Ratio = (*I*_Sum_ − *I*_Tm_)/*I*_Tm_. Furthermore, the UCL-NCs probe could apply in vivo to monitor intratumoral temperature, and the thermal sensitivity of the hybrid system reached 5.6%/K (Figure 12a) [89].

Dynamic temperature mapping in real-time is a powerful technology for wide-field photoluminescence lifetime imaging. Liang et al. designed a single-shot photoluminescence lifetime imaging thermometry (SPLIT), utilizing NaGdF_4_: Er^3+^,Yb^3+^@NaGdF_4_ nanoparticles as indicators. The lifetime of Er^3+^ red emission was more sensitive to temperature than the green emission, resulting from the larger energy separation between emitting and the lower-lying exciting states. Applied in longitudinal temperature monitoring successfully by overlaying chicken breast tissue, the SPLIT proved to be independent with tissue thickness and excitation light power density. When applied in a single-layer onion epidermis sample for single-cell temperature mapping, the lifetime/temperature maps were recorded in 3 s measurement window (Figure 12b). The SPLIT was demonstrated to be resilient to spatial intensity variation, while being advantageous in handling temporal intensity variation [90].

The luminescence lifetime imaging in the NIR could be used for temperature sensing. In the experiment of Soga, the temperature was found to be independent with meat depth from 0mm to 1.4 mm and luminescence intensity [63]. Li et al. designed a Nd-Yb co-doped nanothermometer to detect the temperature in vivo. The Nd^3+^ ions possess thermally rich energy levels to assemble responsive energy for the endogenous relative thermal response. In this structure, Yb^3+^ as energy acceptor was packed closely with Nd^3+^ in a nanocrystal with a diameter of 11 nm, owning tunable intensity and lifetime. The various lifetimes of Yb^3+^ (975 nm) corresponding to different temperatures could be observed due to energy transfer from ^4^F_5/2_ states of Nd^3+^ to Yb^3+^ under 793 nm excitation. The long circulation PEG modified nanoparticle was injected into a living mouse. After its footpad was stuck on a heat/cool pad through 793 nm excitation, the temperature difference could be distinguished between artery and vein, resulting from thermal relaxation. After stopping the heating process, the value of temperature loss was 10% in artery, and 25% in vein after 20 min. The lifetime of the optimized probe exhibited an excellent temperature sensitivity of 0.27 K in vivo [91].

Another nanothermometer was designed for sensing temperature utilizing the NIR-II luminescence lifetime of Yb^3+^ at 1000 nm, which is sensitive to temperature at different tissue depths. The NaYF_4_@NaYF_4_: Yb^3+^,Nd^3+^@CaF_2_ with a size of 13.5 nm acted as a probe for detecting temperature in vivo. Due to back energy transfer from Yb^3+^ to Nd^3+^ and energy migration among Yb^3+^ ions, the doping concentration of Nd^3+^ and Yb^3+^ could affect the temperature sensitivity. NIR-II lifetime-encoded images are acquired by a NIR-sensitive InGaAs camera due to the precisely defined delay time set by the square-wave pulsed excitation laser. The luminescence lifetime versus temperature was calibrated by measuring a series of lifetime-hued images of nanoprobe solution. When the nanoprobes were injected into the inflamed and normal mouse, it exhibited a temperature difference of 2.3 °C according to a thermal camera. In addition, it showed a high-temperature sensitivity of 1.4–1.1% °C^−1^ with the biological tissue up to 4 mm, ranging from 10 °C to 64 °C. The nanothermometers could diagnose murine inflammation in vivo based on lifetime responsiveness to temperature, and map the temperature distribution in the nanoparticle-probed area (Figure 12c) [66].

### 3.7. pH Sensing

**Figure 13:**
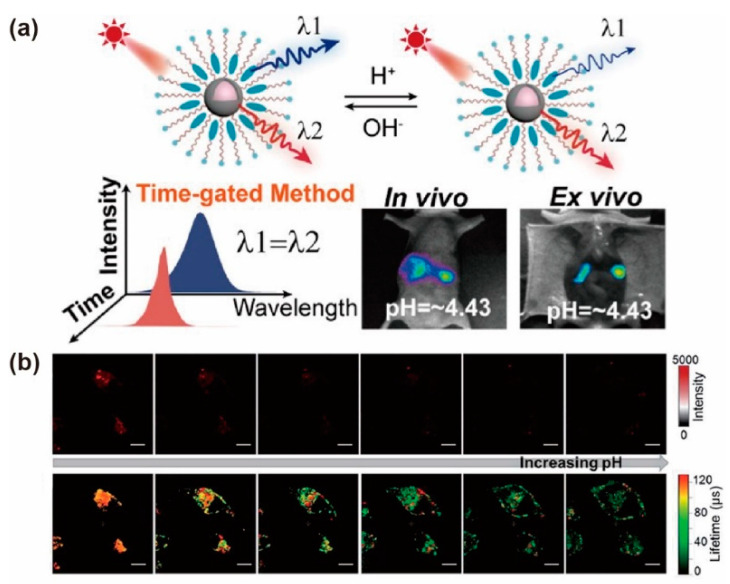
(**a**) The schematic representation and mechanism of the composite probe reversibly responds to variation in the microenvironment and emits two luminescence emissions with different lifetimes. The images of the gastrointestinal area with different pH values in vivo or ex vivo. Reproduced with permission from [93]. Copyright 2020, American Chemical Society. (**b**) The confocal luminescence images (top) and time-resolved luminescence lifetime images (bottom) of F-Yb (10 μM, 4 h) in HeLa cells reacted with 0.5 μM chloroquine. Reproduced with permission from [94]. Copyright 2019, Royal Society of Chemistry.

It is well known that the pH-responsive fluorescein attached to UCNPs could detect pH by the ratiometric responses of emission intensity. Recently, a similar composite probe has been developed, which utilized the distinguished lifetime with the same emission wavelength to monitor a dynamic biological process [92]. Li et al. designed a NaYF_4_: 1%Tm@NaLuF_4_ and Rh760 (pH-responsive dye) composite to detect pH. UCNPs and dye molecules could emit luminescence at 800 nm under the 690 nm excitation, and the lifetimes were 695 μs and 1.40 ns, respectively. Moreover, the pH variation has a negligible effect on the lifetime in NaYF_4_: 1%Tm@NaLuF_4_ nanoparticles, but has a significant impact on the lifetime of Rh760. The time-gated technology could collect the long lifetime (UCNPs at 800 nm), while the short lifetime (Rh760 at 800 nm, only the level of ns) was blocked. Utilizing the time-gated sensing method, they developed a ratio signal of *F*_steady-state_/*F*_time-gated_ to detect pH. For example, they could sensitively monitor the pH value variation from 1.51 to 7.00 in 96-well plates covered with pork tissue, and proved the ratiometric lifetime was reversible and independent of nanoprobe concentration and excitation power density. Similarly, they successfully monitored the gastrointestinal pH value changes in in vivo experiments (Figure 13a) [93].

Designing an NIR lifetime (τ) probe (900–1700 nm) with different pH-responsive lifetimes is a synthetic challenge. Recently, Zhang et al. designed Yb^3+^ porphyrinate (F-Yb) as a pH-sensitive molecular probe. NIR emission and lifetime of Yb^3+^ increased with pKa values of ca. 6.6 from pH 9.0 and 5.0. The lifetime at 1000 nm was prolonged from 135 μ to 170 μs with pH from 5.0 to 1.0 due to reduced exposure to water and aggregation. Oral gavage experiments in nude mice were performed through the NIR τ probe F-Yb for in vivo pH detection. The lifetime of F-Yb in the stomach is 170 μs and that in the intestine is 110 μs. The pH values were 1.5 and 6.0 in the stomach and intestine, respectively. As a result, different organs could be distinguished with quantitative readouts by using the NIR τ probe F-Yb in time-resolved fluorescence lifetime imaging (Figure 13b) [94].

## 4. Conclusions and Outlooks

Compared with traditional fluorescence detection, lifetime sensing based on the lanthanide nanomaterials is not susceptible to tissue thickness, laser power intensity, background interference and solution concentration. Furthermore, the signal can be enhanced by methods such as time-gated lifetime imaging, ratiometric detection and lifetime-coded technology. While the strategies based on measuring the lifetime signal changes in lanthanide-doped nanomaterial for biodetection and bioimaging have promising prospects, there exist many technical challenges to be overcome in practical application. For example, the nanoprobes’ luminescence intensity should be further increased to improve the lifetime signal acquisition speed. It can be solved by optimizing the concentration of dopant, core–shell structure and antenna addition. Moreover, it is highly desirable to increase the stability in the biological microenvironment. Numerous composite nanoprobes consist of lanthanide nanoparticles and molecules such as dye, fluorescein, etc., which are susceptive to their surroundings. Hence, it is necessary to develop stable nanoprobes and measurement techniques. Last but not least, the use of lifetime-based sensing technology could be extended to develop multimodality nanoplatforms with synergistic effects for diagnosis and treatment [95]. This technology will draw wider attention to the development trajectory of fundamental research and clinical applications in luminescent materials.

## Data Availability

Data are contained within the article.
